# Leiomyosarcoma arising in irradiated region after breast-conserving surgery: a case report

**DOI:** 10.1186/s40792-015-0072-y

**Published:** 2015-09-07

**Authors:** Satoshi Hayashi, Masahiro Kitada, Yoshinari Matsuda, Kei Ishibashi, Nana Takahashi

**Affiliations:** Breast Center, Asahikawa Medical University, Midorigaoka-Higashi 2-1-1-1, Asahikawa, Hokkaido 078-8510 Japan

**Keywords:** Leiomyosarcoma, Post-irradiation sarcoma, Breast cancer, Radiation therapy

## Abstract

**Background:**

Radiation therapy (RT) is considered a risk factor for the development of sarcoma in patients with breast cancer. However, there are few reports regarding post-irradiation sarcoma (PIS).

**Case presentation:**

The patient was a 59-year-old woman who presented with a chief complaint of induration in the lower outer quadrant of the left breast. She underwent breast-conserving surgery (BCS) for breast cancer located in the left upper inner region and received endocrine therapy following RT (50 Gy/25 fractions/5 weeks) for breast conservation 6 years previously. Core needle biopsy revealed leiomyosarcoma. There was no distant metastasis. Repeat BCS including part of the pectoralis major muscle was performed. Chest wall resection was not performed because of the lack of invasion. Based on the morphological and immunohistochemical features, a diagnosis of leiomyosarcoma was made. All of the resection margins in the specimen were tumor-free. She has been disease-free for over 20 months.

**Conclusions:**

Post-irradiation leiomyosarcoma is an extremely rare tumor with high malignant potential, and thus, multidisciplinary therapy and close follow-up are advised.

## Background

The combination of breast-conserving surgery (BCS) and radiation therapy (RT) has become a standard treatment for breast cancer. Adverse events are occasionally observed following RT, and post-irradiation sarcoma (PIS) is one of the serious events. Some reports have documented the occurrence of post-irradiation angiosarcoma. However, few reports have described post-irradiation leiomyosarcoma of the breast. We report a case of a post-irradiation leiomyosarcoma that developed following BCS with RT.

## Case presentation

**Fig. 1:**
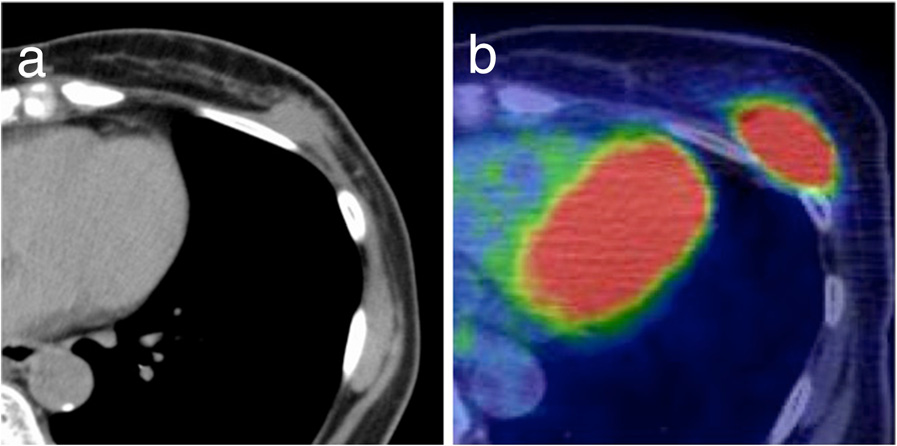
Imaging findings. On positron emission tomography (PET), the maximum standardized uptake value was 12.7, which corresponded to the tumor finding on computed tomography (CT). **a** CT. **b** PET

**Fig. 2:**
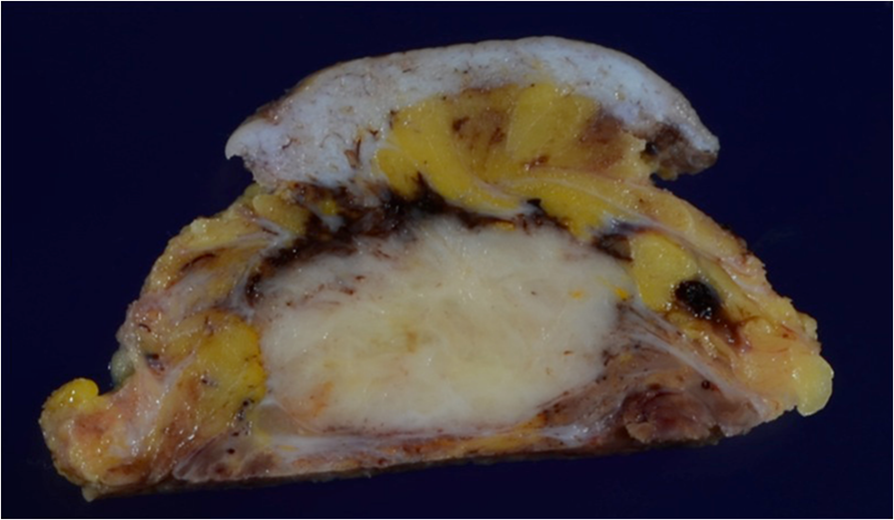
Macroscopic appearance of the resected tumor. The tumor was solid and grayish with smooth margins

**Fig. 3:**
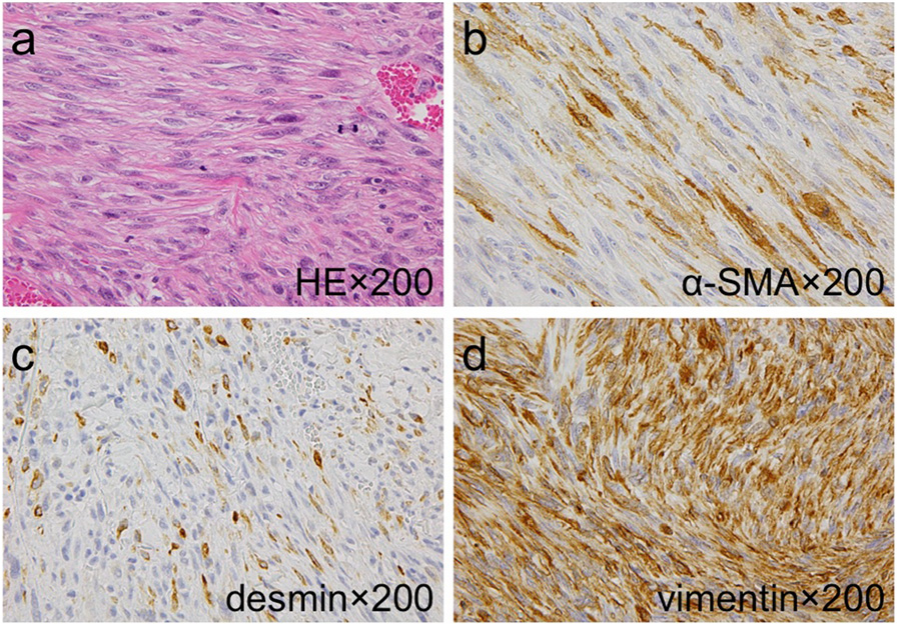
Pathological findings (magnification ×200). The tumor was a 30 × 23 mm tumor with atypical spindle cells arranged in a fascicular histoid pattern. Immunohistochemistry demonstrated that the tumor cells were positive for α-smooth muscle actin (α-SMA), desmin, and vimentin. **a** Hematoxylin-eosin staining. **b** α-SMA. **c** Desmin. **d** Vimentin

A 59-year-old woman was referred to our hospital with a lump located in the lower outer quadrant of the left breast. She underwent BCS as well as endocrine therapy and RT (50 Gy/25 fractions/5 weeks) delivered to the residual breast for breast cancer of the left breast upper inner region 6 years previously. Clinical examination revealed a 20-mm firm lump fixed to the underlying pectoralis major muscle. The overlying skin was free of tumor. There was no palpable axillary lymph node. Mammography revealed a focal asymmetrical density in the retromammary space. Ultrasonography revealed a lobulated and well-defined mass (17.5 × 16.4 × 10.6 mm in size) with relatively hypoechoic, heterogeneous internal echoes. Core needle biopsy (CNB) of the lump suggested atrophic mammary glands. The tumor had increased in size after 3 months of follow-up. Re-CNB was performed, and pathological diagnosis was leiomyosarcoma. On positron emission tomography with ^18^F-fluorodeoxyglucose, the maximum standardized uptake value was 12.7, which corresponded to the tumor finding on computed tomography (Fig. 1a, b). The tumor was limited to the breast and pectoralis major muscle, and there was no distant metastasis. Biochemical blood examinations did not reveal abnormalities. She underwent local excision of the mass with a 2-cm margin including the skin and pectoralis major muscle under general anesthesia. The macroscopic specimen was solid and grayish with smooth margins (Fig. 2). Postoperative histopathology revealed a 30 × 23 mm tumor with atypical spindle cells arranged in a fascicular histoid pattern (Fig. 3a). Immunohistochemistry demonstrated that the tumor cells were positive for α-smooth muscle actin (α-SMA), desmin, and vimentin (Fig. 3b–d). Based on the morphological and immunohistochemical features, the tumor was diagnosed as leiomyosarcoma. Our findings for this case corresponded with the criteria for PIS [1]. All of the resection margins were free of tumor. She has been alive for 20 months without recurrence.

## Conclusions

We reported a case of post-irradiation leiomyosarcoma that developed following BCS. The incidence of PIS may continue to increase because of the combined use of BCS and RT as a standard therapy for breast cancer. There are some prior reports regarding PIS. However, most of these reports described post-irradiation angiosarcoma instead of leiomyosarcoma. Yap et al. [2] reported that among sarcomas occurring within the RT field, angiosarcoma accounted for 56.8 % of the lesions, with malignant fibrous histiocytoma (MFH) accounting for 15.9 %, fibrosarcoma and chondrosarcoma accounting for 6.8 %, and leiomyosarcoma accounting for 4.5 % of the lesions.

The latency period between RT and the appearance of PIS ranges from 4 to 36 years with a mean of 12 years, and the average radiation dose was 53 Gy [3]. There was no difference between patients treated with megavoltage radiation and those treated with orthovoltage radiation concerning the type of sarcoma, its location, or survival rates [4]. This means that the occurrence of PIS is a stochastic effect instead of a deterministic effect.

PIS is often difficult to diagnose in patients with fibrosis legions after RT, and thus, the diagnosis may occur late [3]. In our case, we could diagnose post-irradiation leiomyoma after re-biopsy. Therefore, it may be necessary to perform excisional biopsy to exclude the possibility of PIS.

Appropriate morphological features should distinguish leiomyosarcoma of the breast. In addition, immunohistochemical analysis is a useful diagnostic tool. The differential diagnosis of leiomyosarcoma includes various types of sarcoma, such as fibrosarcoma, MFH, and phyllodes tumor. In leiomyosarcoma, tumor cells are positive for α-SMA, desmin, and vimentin and negative for cytokeratin, epithelial membrane antigen, and S-100 [5]. In our case, immunohistochemistry revealed that the tumor cells were positive for α-SMA, desmin, and vimentin.

Among the treatment options for primary leiomyosarcoma, surgery is generally considered the primary treatment [6]. In contrast, des Guetz et al. reported that in a neoadjuvant therapy group of 19 patients who received chemotherapy, nine patients achieved a clinical partial response [3]. In addition, two post-irradiated histologic subtypes, leiomyosarcoma and undifferentiated sarcoma, were associated with greater clinical responses than the remaining subtypes [3]. These data suggest that chemotherapy should be considered in the treatment of PIS, in contrast to primary sarcomas. We have to include both repeat surgery and chemotherapy in the treatment plan in cases of recurrence of distant metastasis.

The prognosis for patients with PIS is reported to be dismal, with a median survival time ranging from 2.3 [2] to 2.6 years [3], and the 2-year survival rate has been reported as 32 % [4]. However, survival was much better in patients who responded to neoadjuvant chemotherapy and those who underwent surgery.

We reported the first case of post-irradiation leiomyosarcoma of the breast in Japan. In addition, this malignancy is rare globally. However, the lesion in our patient may have been primary leiomyosarcoma, and leiomyosarcoma of the breast is extremely rare. Further research is required. Both surgery and chemotherapy are important treatment options for post-irradiation leiomyosarcoma, and close follow-up are advised.

## Consent

Written informed consent was obtained from the patient for publication of this case report and any accompanying images. A copy of the written consent is available for review by the Editor-in-Chief of this journal.

## Abbreviations

α-SMA: α-smooth muscle actin
BCS: Breast-conserving surgery
CNB: Core needle biopsy
MFH: Malignant fibrous histiocytoma
PIS: Post-irradiation sarcoma
RT: Radiation therapy
